# Conference Report: The FutuRE oF MinimalLy InvasivE GI and Capsule DiagnosTics (REFLECT), September 2025

**DOI:** 10.3390/diagnostics16091315

**Published:** 2026-04-27

**Authors:** Alexandra Agache, Niels Gellert Olesen, Asta Slott Skifte, Jakob Frederik Frøkjær Justsen, Anastasios Koulaouzidis

**Affiliations:** 1 Department of Surgery, Odense University Hospital, 5700 Svendborg, Denmark; niels.gellert.olesen@rsyd.dk (N.G.O.); asta.slott.rasmussen@rsyd.dk (A.S.S.);; 2 Department of Clinical Research, University of Southern Denmark, 5000 Odense, Denmark; 3 Department 10 General Surgery, University of Medicine and Pharmacy ‘Carol Davila’, 050474 Bucharest, Romania; 4 Department of Gastroenterology, Pomeranian Medical University, 70-111 Szczecin, Poland

**Keywords:** conference, minimally invasive diagnostics, gastrointestinal imaging

## Abstract

Capsule endoscopy (CE) is evolving from a primarily small-bowel imaging modality into a broader diagnostic platform that increasingly incorporates artificial intelligence (AI), robotic technologies, biosensing capabilities, and decentralized models of care. The REFLECT symposium brought together an international, multidisciplinary audience of clinicians, engineers, scientists, and healthcare stakeholders to critically evaluate the present and future role of CE across a range of gastrointestinal (GI) applications, including inflammatory bowel disease, GI bleeding, coeliac disease, and colorectal cancer screening. Discussions explored the clinical impact of panenteric and colon capsule endoscopy, the potential of AI to enhance diagnostic performance and streamline workflows, innovations in capsule hardware, and the design of patient-centred diagnostic pathways. While conventional endoscopy continues to serve as the benchmark in many clinical scenarios, CE was recognized for its ability to improve access, acceptability, and scalability when deployed in appropriately selected populations. The symposium also identified key barriers to broader implementation, such as reinvestigation rates, absence of standardized quality indicators, limited real-world evidence for AI tools, and ongoing economic and environmental challenges. Overall, the meeting highlighted the importance of gradual, evidence-driven integration of CE, supported by robust validation, standardized metrics, close clinician-engineer collaboration, and meaningful incorporation of patient experience, to support the development of a safe, equitable, and sustainable pathway.

## 1. Introduction

The sixth edition of the REFLECT symposium—the futuRE oF MinimalLy InvasivE GastroIntestinal and Capsule DiagnosTics—brought together clinicians, scientists, engineers, industry partners, and health-system stakeholders to critically examine the current and future role of capsule endoscopy (CE) in clinical practice. Through a series of thematic sessions, the meeting explored the application of CE in inflammatory bowel disease (IBD), gastrointestinal (GI) bleeding, coeliac disease, colorectal cancer screening, and community diagnostic pathways, alongside emerging innovations in capsule hardware, robotic control, non-imaging sensing, and artificial intelligence (AI)-driven interpretation. Particular attention was given to the challenges of translating technological advances into clinically meaningful outcomes, including patient selection, validation standards, cost-effectiveness, environmental sustainability, and ethical considerations surrounding trustworthy AI.

Rather than focusing solely on technological novelty, the symposium emphasized the need for stepwise and evidence-based adoption of CE, aligned with clinical workflows and patient needs. Discussions highlighted the importance of standardized quality metrics, real-world validation of AI tools, integration of patient-reported outcomes, and collaboration between clinicians and engineers to ensure that innovation delivers tangible clinical benefit. This article summarizes the key discussions, debates, and conclusions from the symposium, providing an overview of the current state of CE and outlining future directions required to support its safe, equitable, and sustainable implementation in GI care.

## 2. Session I

The first session was covered by the iCare group members who brought into attention the role of CE in inflammatory bowel disease and celiac disease and whether panenteric capsule endoscopy (PCE) could assume an earlier role in the diagnostic algorithm for GI bleeding.

CE was highlighted as a central tool for assessing small-bowel and panenteric disease activity in Crohn’s disease (CD), particularly in the context of treat-to-target strategies and mucosal healing. CE is the most sensitive modality for detecting proximal small-bowel inflammation, revealing residual lesions even in patients in clinical remission, where mild inflammation predicts relapse [1,2]. Although treat-to-target evidence remains mixed—positive outcomes in CALM (2017) [3] and REACT (2015) [4], but not in REACT-2 (Jairath V, UEG Week 2022) [5] or STARDUST [6]—emerging data support proactive CE-guided management. Notably, Ben-Horin et al. (2019) demonstrated that risk stratification using the Lewis Score (>350) and proactive escalation significantly reduced clinical flares over 24 months, representing the first CE-based treat-to-target study [2]. In pediatrics, CE offers a sedation and radiation-free, minimally invasive approach particularly suited to CD, where small-bowel involvement is common and frequently underdiagnosed without CE [7,8]. CE changes management in over half of pediatric cases and is essential for diagnosis, disease classification, and extent assessment, except in typical ulcerative colitis (UC) [7]. CE and MRE were emphasized as complementary—mucosal versus transmural healing—given documented discrepancies between the two [9]. Despite limitations such as retention risk, incomplete exams, and lack of biopsy capability, current ECCO-ESGAR-ESP-IBUS guidelines endorse broader CE use, with growing evidence supporting panenteric capsules in both adult and pediatric IBD [10,11,12,13].

The symposium concluded that CE should not be used routinely in coeliac disease, but has clear value in selected and complicated cases, where it enables noninvasive, pan–small-bowel assessment to exclude malignancy, define disease extent, and support timely intervention. CE may reduce the need for repeated upper endoscopy in tertiary centers and assist in risk stratification, as the severity of villous atrophy is associated with adverse outcomes [14,15]. While limitations remain—particularly the inability to obtain histology and the patchy nature of mucosal injury—advances in AI are expected to improve detection, grading of villous atrophy, and monitoring of emerging therapies, positioning CE as an important complementary tool rather than a replacement for conventional endoscopy [16,17].

Conventional endoscopy remains the recommended first-line approach for GI bleeding, offering the strongest evidence for timely diagnosis and therapy, particularly when performed within 6–24 h for upper GI bleeding. While PCE shows promise for earlier, noninvasive evaluation and may reduce unnecessary colonoscopies in selected patients, its current use is limited by preparation complexity, incomplete examinations, long reading times, and lack of therapeutic capability. ESGE guidelines emphasize patient stabilization, risk stratification, and appropriate use of colonoscopy and CT angiography, supporting continued adherence to established algorithms while encouraging further research to identify patient subgroups that may benefit from earlier PCE integration, especially as artificial intelligence advances improve feasibility [18,19].

The session ended with a discussion about an integrated, community-delivered CCE pathway that was shown through preliminary assessment of an ongoing project, to be feasible, safe, and effective in expanding access, reducing diagnostic backlogs, and improving patient experience when applied to carefully selected low-risk patients. Early outcomes demonstrate rapid reporting, low conversion to invasive testing, no capsule retentions, and potential environmental benefits, while future adoption depends on scaling the model, standardizing CCE-specific quality indicators, and generating robust cost-effectiveness evidence to support wider implementation within the National Health Service framework [20].

## 3. Session II—Innovative Platforms and Clinical Trials

Ongoing clinical trials and innovative platforms took the spotlight and highlighted AI as a critical enabler for the scalability and clinical viability of CCE, addressing key limitations related to reader fatigue, prolonged interpretation time, diagnostic variability, and cost-effectiveness. Data from the CESCAIL project demonstrated that AI-assisted CCE significantly improves sensitivity, negative predictive value, and overall diagnostic accuracy for polyp detection—particularly for left-sided, small (<10 mm), and sessile lesions—while reducing physician reading time more than fivefold, albeit at the expense of higher false-positive–driven colonoscopy conversion [21]. Complementary projects (ColoCap, DanCap) emphasized that high reinvestigation rates remain the principal barrier to economic sustainability, underscoring the importance of optimized patient selection, bowel preparation, and clearer definitions of clinically relevant findings [22]. Finally, large-scale initiatives such as SEARCH (Synthetic hEalthcare dAta goveRnanCe Hub) illustrated how federated learning, synthetic data, and explainable AI may support robust clinical decision support systems, training, and outcome prediction, reinforcing that AI-human collaboration—rather than automation alone—will define the future role of CCE in colorectal cancer screening and surveillance [23].

## 4. Session III—Capsule Hardware & Pre/Clinical Studies

The session underlined the rapid innovation in capsule platforms beyond imaging, emphasizing controllable vibro-impact capsule robots, biosensing technologies, and standardized performance metrics as foundations for future clinical adoption. Vibro-impact capsules demonstrated proof-of-concept potential for nonvisual tissue characterization, using capsule–tissue dynamic responses to discriminate normal from abnormal tissue with very high accuracy in preclinical models, while underscoring the need for controlled locomotion and human validation [24]. Broader discussions stressed that successful translation of such technologies requires careful progression along technology-readiness levels through close clinician–engineer collaboration [25]. In parallel, the session called for standardized definitions of post-CCE colorectal cancer (pCCECRC), aligned with colonoscopy quality frameworks, to enable meaningful benchmarking and KPI development. Finally, emerging non-imaging capsules—including biochemical, electrochemical, impedance, and microbiome-sensing devices—were presented as promising tools to interrogate inflammation, metabolism, motility, and gut–brain signaling, although significant challenges remain related to biofouling, power consumption, localization, and data robustness [26,27].

## 5. Session IV—Autocapsule

It presented how rapid advances in microelectronics, robotics, wireless power transfer, and sensing technologies are driving a new generation of low-cost, high-functionality autonomous capsules aimed at improving comfort, accessibility, and diagnostic yield of GI investigations. The AUTOCAPSULE project demonstrated that leveraging commodity semiconductor technologies can dramatically expand capsule capabilities—robotic locomotion, real-time localization, micro-ultrasound, and biochemical sensing—while driving costs down, enabling at-home or community-based diagnostics [28,29]. Magnetic robotic capsules and flexible magnetic endoscopes showed early human feasibility with minimal discomfort and precise navigation, while ingestible redox-sensing capsules provided stable in vivo measurements of pH and oxidative stress without bowel preparation, suggesting new avenues for monitoring inflammation and gut health [30,31]. Progress in ultrasound CE and wireless power/data transfer underscored both the promise of subsurface tissue characterization and the persistent challenges of power consumption, miniaturization, localization, and data bandwidth [28,29,32]. Across all technologies, the session emphasized that successful translation depends on stepwise clinician–engineer collaboration, clear clinical use cases, and aligning technological innovation with meaningful patient benefit.

## 6. Session V—AICE Project

The AICE project (AI-Supported Image Analysis in Large Bowel Camera Capsule Endoscopy) represents an end-to-end, AI-enabled ecosystem for CCE, integrating patient selection algorithms, digital patient engagement (PROMs/PREMs), secure cloud-based data infrastructure, and clinically interpretable AI decision-support tools to enable scalable, cost-efficient diagnostics [33,34]. The project highlights that effective AI deployment in CCE depends as much on optimized patient selection, coaching, bowel preparation, reporting standards, and outcome tracking as on algorithmic performance, with external validation and explainability remaining critical challenges [34,35]. Patient experience emerged as a central determinant of pathway success, with evidence that CCE is perceived as less invasive and more acceptable than optical colonoscopy, while bowel preparation and inadequate education remain key barriers—addressed through co-designed digital companion apps [35]. Across both colon and small-bowel CE, AI shows strong potential to markedly reduce reading times and improve lesion detection, but consensus emphasized the need for standardized ground truth, real-world validation, clear clinical responsibility, and trustworthy governance before routine implementation.

**The closing session, the round table** emphasized the growing gap between clinical needs and technological possibilities, centering on equity, sustainability, validation, and practicality in CE. Participants debated whether CE should be offered to populations disengaging from colonoscopy due to cultural, religious, or gender-related barriers, acknowledging its potential to improve equity while recognizing current cost constraints and concerns about overextending healthcare resources.

Clinicians emphasized the need for simple, actionable outputs rather than novel or complex technical “languages,” while accepting that learning new paradigms is possible if clinical value is clear. Environmental sustainability emerged as an unavoidable issue, with endoscopy recognized as resource-intensive; however, patient safety and clinical effectiveness were consistently prioritized over carbon reduction, with prevention and reduced procedure volumes viewed as the most impactful solutions. There was broad agreement that validation requirements should be proportional to clinical risk, with strong support for real-world data—particularly for AI systems—alongside controlled studies. The discussion also acknowledged that wider CE adoption may challenge traditional endoscopy unit business models, though therapeutic endoscopy would remain central and capsule costs are expected to fall with scale. Finally, optimizing bowel preparation was seen as a solvable, high-impact factor, with correct timing and patient compliance—rather than choice of regimen—identified as the most critical determinants of successful CE examinations.

## 7. Conclusions

CE is most valuable when applied selectively, not universally. In CD, CE improves detection of proximal and panenteric inflammation and can support treat-to-target strategies when guided by risk stratification. In GI bleeding and coeliac disease, conventional endoscopy remains first-line, but CE adds value in defined subgroups and complicated cases. In colorectal diagnostics, CCE is feasible and effective in low-risk, FIT-negative, or capacity-constrained populations, particularly when delivered in community settings. AI is an enabler, not a substitute; AI consistently improves diagnostic accuracy and dramatically reduces reading time in both small-bowel and colon CE. False positives, interpretability, ground truth definition, and real-world validation remain key barriers. Human–AI collaboration is essential; clinical responsibility ultimately remains with the clinician. Patient experience and equity matter. CE is perceived as less invasive, less intimidating, and more acceptable than colonoscopy, lowering psychological and cultural barriers to screening. Digital education, coaching, and companion apps are critical to improve bowel preparation, compliance, and outcomes. Equity should be addressed pragmatically through targeted pathways, not indiscriminate adoption. Technology is moving faster than clinical frameworks: Robotic capsules, wireless power, ultrasound capsules, and biosensing platforms show strong preclinical promise but require clear clinical use cases. Technology readiness levels must guide development to avoid clinically irrelevant innovation. Common language and shared priorities between clinicians and engineers are essential. Sustainability and cost cannot be ignored. Endoscopy has a significant environmental footprint; CE may offer greener alternatives in selected scenarios. Cost-effectiveness of CCE hinges on reducing reinvestigation rates, improving bowel preparation, and defining clinically meaningful findings. Volume, scale, and patient selection will drive cost reduction more than technology alone.

## 8. Summary

The conference underlined that CE is still transitioning from a niche diagnostic modality to a platform technology spanning diagnostic, monitoring, robotics, biosensing, AI and community-based care.

While conventional endoscopy remains the reference standard in many indications, CE offers clear advantages in patient acceptability, equity, scalability, and sustainability. However, widespread adoption is currently limited by variability in evidence, lack of standardized quality metrics, high reinvestigation rates, incomplete validation of AI tools, and insufficient alignment between technological innovation and clinical workflows.

The future of CE is not defined by replacing conventional endoscopy, but by doing the right test for the right patient at the right time, supported by trustworthy AI, patient-centered design, and sustainable healthcare principles. The symposium underscored that technological readiness alone is insufficient—clinical relevance, equity, and evidence must lead innovation.

## Data Availability

No new data were created or analyzed in this study.
